# Rater Training in Medical Education: A Scoping Review

**DOI:** 10.7759/cureus.11363

**Published:** 2020-11-06

**Authors:** Ashley Vergis, Caleb Leung, Reagan Roberston

**Affiliations:** 1 Surgery, St. Boniface Hospital, University of Manitoba, Winnipeg, CAN

**Keywords:** rater training, medical education, technical skills, non-technical skills, clinical assessment tools

## Abstract

There is an increasing focus in medical education on trainee evaluation. Often, reliability and other psychometric properties of evaluations fall below expected standards. Rater training, a process whereby raters undergo instruction on how to consistently evaluate trainees and produce reliable and accurate scores, has been suggested to improve rater performance within behavioral sciences. A scoping literature review was undertaken to examine the effect of rater training in medical education and address the question: “Does rater training improve performance attending physician evaluations of medical trainees?” Two independent reviewers searched PubMed®, MEDLINE®, EMBASE™, the Cochrane Library, CINAHL®, ERIC™, and PsycInfo® databases and identified all prospective studies examining the effect of rater training on physician evaluations of medical trainees. Consolidated Standards of Reporting Trials (CONSORT) and Strengthening the Reporting of Observational Studies in Epidemiology (STROBE) checklists were used to assess quality. Fourteen prospective studies met the inclusion criteria. All had heterogeneity in design, type of rater training, and measured outcomes. Pooled analysis was not performed. Four studies examined rater training used to assess technical skills; none identified a positive effect. Ten studies assessed its use to evaluate non-technical skills: six demonstrated no effect, while four showed a positive effect. The overall quality of studies was poor to moderate. Rater training in medical education literature is heterogeneous, limited, and describes minimal improvement on the psychometric properties of trainee evaluations when implemented. Further research is required to assess rater training’s efficacy in medical education.

## Introduction and background

In many fields, including medicine, measuring performance is limited to subjective observational judgments. Recent changes to traditional medical education present new challenges for training physicians. Initiatives towards competency-based training have caused many programs to introduce the use of standardized, outcomes-based clinical assessment tools. However, the psychometric properties of these tools remain insufficient for high-stakes testing, with reliability often below desired benchmarks. Although several means to improve reliability exist, many studies fail to suggest or examine these options. One method to improve the reliability of assessments is to attempt to improve the objectivity of raters [1].

Rater training (RT) is a process whereby raters undergo instruction on how to evaluate trainees best and produce reliable and accurate scores. RT was developed in an effort to address the natural bias introduced by subjective performance assessments. There is compelling evidence in the behavioral and social sciences literature to suggest that RT can improve rater performance [2]. The process is thought to work by focusing on optimizing the standardized use of a tool and limiting the effect of individual preconceived notions [3]. RT is commonly used in these disciplines to improve the psychometric properties of a variety of observational assessment tools [1-6]. In a landmark study on RT methods, Woehr and Huffcutt classified RT into four different types, including (1) rater error training, (2) behavioral observation training, (3) performance dimension training, and (4) frame-of-reference training [2].

Rater error training educates raters regarding common rating errors such as halo, central tendency, and leniency. Evidence of rating errors is generally considered to reflect a considerable inaccuracy degree within an evaluation [2]. Generally, specific errors are defined, and the raters are then given strategies on how to avoid them [7]. For example, raters may be informed to look for both good and bad performance features and avoid forming overall impressions to prevent halo [4]. Behavioral observation training instructs raters to observe and record behavior as opposed to forming global judgments. Raters are taught to anticipate specific behaviors within a dimension and make a careful record of these observations to improve recall of particular events [2, 7]. An example would be classifying a subject based on the exact number of times a specific behavior was present, or action was performed. Performance dimension training educates raters on the specific dimensions used to evaluate trainees before the observation is begun. Understanding each dimension can then guide rater observation and subsequent evaluation. Each dimension is clearly defined, and examples of actions and behaviors associated with the dimension are given. Having raters participate in assessment tool development or familiarizing raters with the tool prior to observation are examples of performance dimension training [2]. The frame of reference (FOR) training builds a common construct between raters, which they use to observe and evaluate subjects. Raters are instructed on performance standards for each dimension. Rating tool dimensions and their associated behaviors are defined, leading FOR training to often include aspects of behavioral observation and performance dimension training. The desired level of performance to each specific rating is explained in order to create a shared definition between raters of an appropriate ranking for an observed performance. In some instances, an element of rater practice, discussion, and feedback is incorporated into the training to further develop the shared criteria [2, 7].

Despite RT's proven effectiveness in other fields, RT has not been widely studied in medical and surgical education. Many studies of commonly used clinical assessment tools have commented on their insufficient psychometric properties, particularly poor reliability [8-10]. Even so, many studies fail to suggest or examine the available options to improve reliability. Existing options include increasing the number of assessments, modifying the tools themselves, or improving rater objectivity, such as through the use of RT. In medicine, significant time constraints for both trainees and evaluators make increasing the number of assessments extremely challenging [11]. Changing existing tools creates problems pertaining to having multiple versions of a similar tool, requiring re-validation of the modified instrument [12]. Therefore, we performed a scoping review to examine what is currently known about the effect of RT on trainee assessments in medical education. 

This review is composed based on the master thesis (Maniar R, The Effect of Rater Training on the Reliability and Validity of Technical Skill Assessments: A Randomized Control Trial. Faculty of Graduate Studies of The University of Manitoba, Department of Surgery; 2016).

## Review

Methods

A search for original publications until January 2020 was performed using PubMed®, MEDLINE®, EMBASE™, the Cochrane Library, CINAHL®, ERIC™, and PsycInfo®. The inclusion criteria of the search were prospective studies with RT for physicians as a primary intervention, where some formal description of the training was given. Additionally, some form of the control group was needed, although it was not limited to any specific type so long as it was present. Studies using pre- and post-training comparisons were eligible, as were studies comparing trained raters to an untrained group. Specific psychometric properties such as reliability or validity had to be specified as an outcome variable. Articles were excluded if the RT intervention was not described, if the subjects undergoing training were not attending physicians, if there was no comparison group, or if an outcome variable was not specified. Review articles were also excluded. The specific search terms were "rater training" AND "medical" OR "surgical education". A manual review of the selected articles’ references was also performed to ensure search completion. Two authors assessed articles for eligibility for inclusion. Any disagreements were resolved by a third author at each stage.

Results

**Figure 1:**
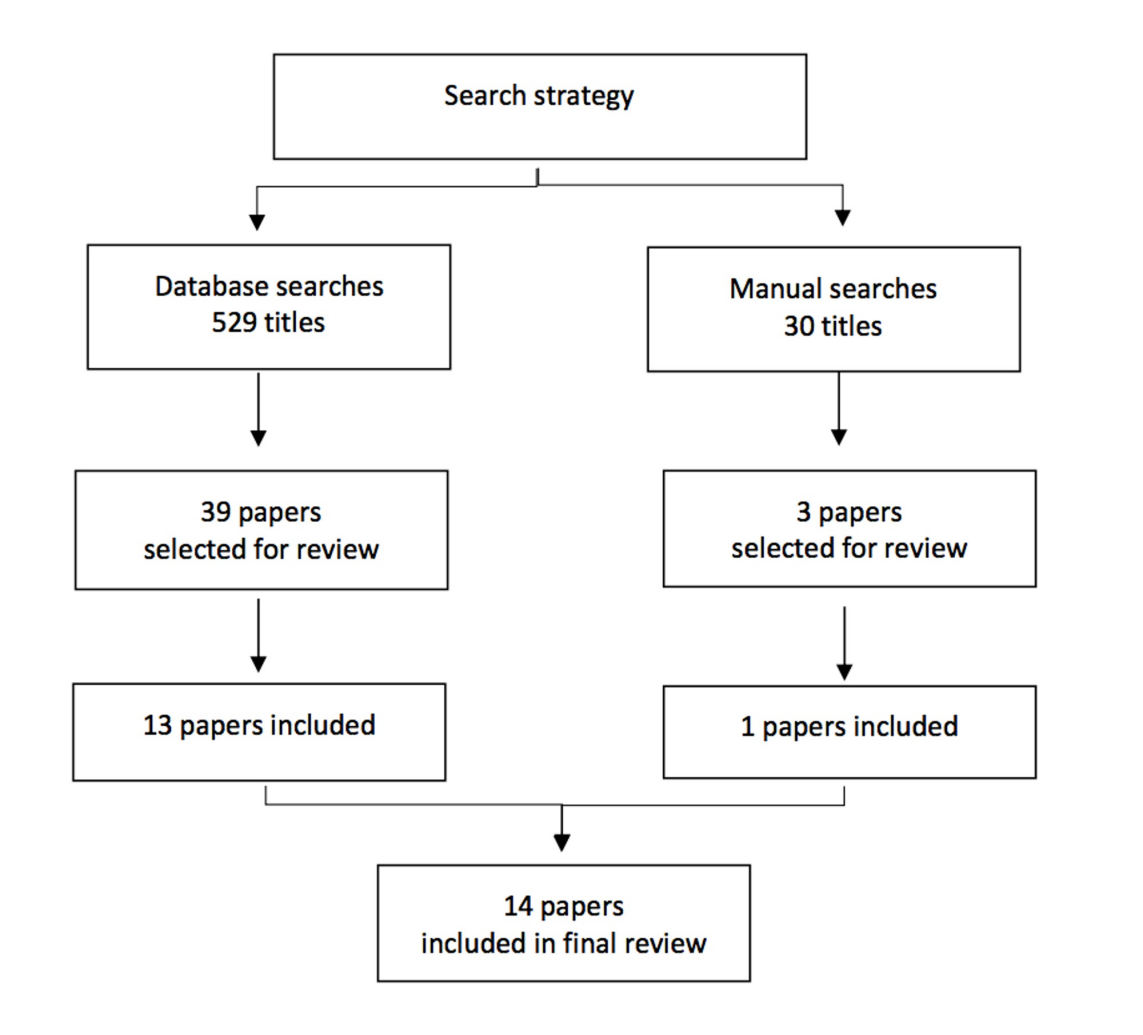
Search strategy for scoping review on rater training in medical education

The initial search strategy found 529 papers for abstract review, with an additional 30 papers found by the manual search. Forty papers were selected for full-text review. Fourteen papers met the criteria and were selected for final inclusion (Figure 1) [10, 11, 13-24].

The included studies with a description of their methods and findings can be found in the Appendix. The quality of included studies was assessed using modifications of the Consolidated Standards of Reporting Trials (CONSORT) and Strengthening the Reporting of Observational Studies in Epidemiology (STROBE) checklists (see Appendix).

Included studies had marked heterogeneity in terms of their design, methods, and type of RT. Ten of the studies were randomized trials, and four were cohort studies. Seven of the studies specifically included surgeons; however, the majority of the studies looked at rating non-technical skills. Only four studies measured assessments of technical skill [13-16]. The majority of the studies had raters evaluating a videotaped clinical encounter. Of these, five used a standardized, scripted encounter as opposed to real patient interactions. Two studies assessed performance in training evaluation reports​​​​​​​ (ITERS), and a final study assessed a surgical oral exam.

The training was in a workshop format for eight of the studies and ranged from an hour-long training session to a four-day course. The remainder of the studies utilized a training video. Although all studies described their RT intervention, only eight studies specified the intervention using well-defined terminology such as “rater error training” or “FOR training”. Types of training ranged from using a single format to incorporating all four types of RT into a workshop. Most studies compared the training group to a group of untrained raters. Two studies compared raters pre- and post-training and George, et al. compared an intensive RT program to an accelerated version [14]. Seven of the studies looked at interrater reliability (IRR), although a variety of statistics were used, including interclass correlations, Cronbach’s alpha, and Kendall's coefficient. The outcomes assessed in the remaining studies included accuracy (four studies), correlation coefficients (one study), and assessment quality (two studies). One study examined the effect of RT on the reliability and validity of the psychometric outcomes.

Rater Training for Technical Skill Assessments

Of the four studies that assessed RT for assessments of technical skill, all were unable to show an effect of training. In the first study by Robertson et al., a FOR training video showed no statistically significant effect on the IRR of forty-seven attending surgeons randomized to RT versus no training and assessing simple suturing and instrument knot tying videos of 10 trainees [15]. The performance was assessed using a procedure-specific checklist, visual analog scale, and a modified Objective Structured Assessment of Technical Skills (OSATS) Global Rating Scale (GRS) [25]. Interclass correlation coefficients (ICC) were measured to assess IRR. Although there was a trend towards improved ICC with RT, this was not statistically significant.

Using the same study population, Robertson et al. also examined the effect of FOR training on the reliability and validity of multiple psychometric assessment tools for surgical suturing and knot-tying, including a pass-fail assessment, a visual analogue scale, a modified OSATS GRS, and a task-specific checklist [16]. Raters were randomized to RT versus no RT. Assessments of trainee suturing and knot-tying videos were made at the initial start of the study and then after a delay of two weeks. Internal consistency and reliability were measured with Cronbach’s alpha and IRR scores. Validity was assessed using univariate and multivariate analyses. Although there was a trend towards improvement of all three domains, there were no statistically significant differences after RT when compared to no RT.

Similarly, in a study by Rogers et al., a rater error training video had no effect on the IRR of eight surgeons evaluating a simple two-handed knot tie video using a standardized checklist [13]. Cronbach's alpha was used to assess IRR. Scores were high for both groups regardless of training (0.80 for the untrained group vs. 0.71 for the trained group), limiting the ability to show a difference between groups. Additionally, rater error training was used, which has been shown to not be the most effective training method, especially for improving reliability. The training group was noted to give more specific comments in their feedback, which the authors suggested may be indicative of some unmeasured effect of training on rater behavior.

The other study assessed technical skills, randomizing surgeon raters to either an accelerated or immersive FOR training session using the Zwisch OR performance GRS [14]. The accuracy of the two groups was measured by comparing scores to an expert consensus score. Although the immersive group had a slightly higher accuracy of 88% as compared to 80% for the accelerated group, this was not statistically significant. There was no difference in the overall Zwisch GRS scores between groups. Although this study was not significant, the difference in accuracy scores was close to achieving significance, suggesting a possible effect of training. The study may have been underpowered to detect a difference as forty-four surgeons were randomized, but only ten were in the non-training group. Accuracy was also assessed using expert consensus scores, which may not be the best measure of a tool’s psychometric properties. Reliability and validity are generally more critical, especially if the tool is to be used for high-stakes purposes.

Rater Training for Non-Technical Skill Assessments

Of the remaining ten studies assessing the evaluation of non-technical skills, four studies had a positive outcome. Holmboe et al. randomized internal medicine attending physicians to a four-day performance dimension and FOR training workshop on using the mini Clinical Evaluation Exercise (CEX) tool [10]. The mini-CEX is an observational instrument that uses GRS to assess a trainee’s clinical interaction with a patient [26]. After training, the tool was used to assess scripted, videotaped clinical encounters. After adjusting for baseline rating and program, the trained group had significant improvement in IRR that persisted for eight months after the workshop [10]. Van der Vleuten, et al. showed a one-hour training session improving the accuracy of medicine attendings using a checklist to evaluate videotaped history and physical skills as compared to those randomized to no training [17]. In two papers by Dudek et al., a home training session for medicine attendings was shown to improve the quality of ITER assessments post-training [18, 19]. The remaining six studies were unable to show an effect of RT [11, 20-24].

Quality of Evidence

The overall quality of the included studies was poor to moderate (see Appendix). Only four of the randomized studies adequately described their randomization techniques, and one was minimally described. Three studies did not explicitly state their eligibility criteria. Only seven studies included a clear and detailed explanation of their training intervention, four had a moderate description, while three studies had only a brief description. Three of the randomized studies did not state if there were any baseline differences between training groups. Three studies failed to list any significant limitations, and in two, this was only very superficially discussed. Finally, only two studies included any type of power calculation, and one of these failed to achieve their desired recruitment. Many of the studies were small and thus may have been underpowered to detect a difference between groups. Notably, the only high-quality study in the group by Holmboe et al. was able to show a significant and prolonged effect of RT [10].

Discussion

Rater training within the social sciences has been demonstrated to have a positive effect on common rating measures. A meta-analysis of 29 comparative studies found moderate effectiveness in all four rater training domains: i.e., rater error training resulted in reduced halo effect or leniency errors, performance dimension training reduced halo error, FOR training improved increased rating accuracy and behavioral observation training results in improved observational accuracy. Among all four domains, FOR training appeared to be the most effective intervention to improve rating accuracy. Although limited in number, several studies also examined the role of combined RT strategies and demonstrated positive effects on various rater errors among small sample sizes [2].

With the continued shift in medical training towards a competency-based medical education and increased evaluation, there is a need to ensure reliable and accurate trainer assessments. Our review found that within the current medical education literature, RT has not been demonstrated to significantly improve various psychometric properties of medical trainee assessments. However, there was a trend towards a positive effect in specific outcomes such as IRR, with the most studied intervention involving FOR training.

One response to the lack of success in improving the psychometric properties of assessments in medical education has been to rethink the approach to assessment. Newer theories advocate moving away from the traditional viewpoint of objective, quantifiable measures as the gold standard in favor of more subjective assessments. Proponents of this model argue the traditional approach is limited because it reduces the evaluation of complex aptitudes of medical trainees into individual quantifiable skills. This results in a loss of overall “gestalt” and limits how to address the evaluation of certain key aspects of modern health care delivery, such as team-based work and collaboration [25]. Others have sought to address the concept of RT within contemporary frameworks by seeking to understand rater error from the perspective of the psychological sciences, specifically impression formation literature. Such theories suggest if rater-based assessments are understood as a psychological and social judgment phenomenon, educators may be better equipped to address and correct the issue of rater error [27].

The decision to focus this review on the traditional psychometric properties of standardized assessments was for two reasons. Firstly, medical education remains focused on quantitative assessment. The advent of competency-based medical education demonstrates this, as it, by definition, seeks to identify and evaluate individual domains required by medical trainees to succeed. Therefore, the continued study and improvement of how evaluations are made and their quality measured are needed, particularly for high-stakes testing. Secondly, technical skills assessment may be most appropriately evaluated by traditional standardized assessments. Other skills, such as clinical decision making or working within team-based healthcare systems, may be less amenable to standardized evaluation forms. However, a technical skill assessment is well suited to this type of measurement. This is reflected by the development, ongoing application, and widespread use of standardized tools such as OSATS in surgical education [25]. Improving the use of these assessment tools within technical fields of medical education may be necessary. For example, by investigating more optimal forms of rater training.

Limitations of our review include marked heterogeneity among studies in terms of the study population, rater training intervention, and measured outcomes. The overall quality of studies was poor to moderate, and no pooled analysis was possible. It is clear that although RT may represent a means to improve the reliability of skill assessments, further high-quality studies are needed to determine the role of RT within medical and surgical education.

## Conclusions

Future research also should investigate the optimal format and duration of rater training for each individual setting or tool. Within the literature examining RT in medical education, there is a lack of evidence on the ideal training format, as there is nearly unlimited variability in the way training can be administered. This makes it difficult to know the best starting point when developing a new training intervention. A variety of training formats have been described, varying from in-person tutorials to training videos, the use of single or multiple RT types, and sessions ranging from less than an hour to multiple day workshops. 

## Appendices

**Table 1:** Summary of studies included in the rater training scoping review BOT - behavioral observation training; FOR – frame of reference training; PDT – Performance dimension training; RET – rater error training; GRS - Global Rating Scale; OSATS - Objective Structured Assessment of Technical Skills; RT - rater training; NOTSS - non-technical skills for surgeons; CEX -  Clinical Evaluation Exercise; CCERR - completed clinical evaluation report rating; ITER - in training evaluation reports;  FITER - final in-training evaluation report; RE - rater error; OR - operating room; ICC - interclass correlation coefficients

Reference	Objective or question	Study design	Setting, population, and n	Intervention	Control	Assessments	Outcome	Comments/results
Robertson et al. 2018 [15] Canada Single centre	Does FOR training improve IRR for evaluations of knot-tying and simple suturing? (five-point GRS, modified OSATS GRS, visual analogue scale)	Randomized controlled (stratified block randomization)	Attending surgeons from multiple specialties; (n=47); voluntary	Seven-minute FOR training video (n=24)	No training (n=23)	10 videos of trainees performing simple suturing and instrument knot tying	IRR – intraclass correlation (ICC) type 2: GRS 0.61 (0.41-0.85) no training vs. GRS 0.71 (0.52-0.89) FOR training three assessment tool measured using mixed-model analysis, showing no differences in mean scores.	Randomized; simple task; short training session; no statistical difference but trends toward higher ICC with RT
Robertson et al. 2020 [16] Canada Single centre	Does RT affect the reliability and validity of four technical skill assessment tools?	Randomized controlled (stratified block randomization)	Attending surgeons from multiple specialties; (n=47); voluntary	Seven-minute FOR training video (n=24)	No training (n=23)	10 videos of trainees performing simple suturing and instrument knot tying with additional assessments at a two-week interval	Trend towards higher reliability (Cronbach’s alpha, IRR) and validity but no statistically significant difference. OSATS GRS appears to be preferred.	Randomized; simple task; short training session; no statistical difference but trends toward improved assessment tools
Rogers et al. 2002 [13] Canada Single centre	Does RT improve IRR for evaluations of med student knot tying (seven-point GRS)	Randomized RT or none; not described how randomized	General surgeons (n=8); numbers in each group not given; voluntary	Rater error training (RET) video (four errors shown three times)	No training	24 videos of real students performing hand tie, rated immediately	IRR - Feldt’s t test on Cronbach’s alpha; no effect (a=0.71 RT vs a=0.80 control)	Small; simple task; short training; high IRR regardless of training
Spanager et al. 2013 [23] Denmark nine centres	Does RT improve reliability rating surgeons non-tech skills (NOTSS; five-point GRS)	Cohort study – pre and post-training	General surgeons; specialists and fellows (n=15); voluntary	Four-hour training workshop (FOR, RET, BOT, PDT)	Pre- and post-training	Nine scripted videos of OR encounters, rated before and immediately after RT	IRR - Cronbach’s alpha (a=0.96 &0.97 pre and a=0.97 & 0.98 post) Pearson’s for construct validity (=0.95)	Non-technical skills; voluntary/assigned participation; non-randomized; no effect
Cook et al. 2008 [22] US Single centre	Effect RT on IRR and accuracy of mini-CEX scores (internal medicine clinical exam) (five-point GRS)	Randomized controlled (21/54 declined randomizing)	Medicine faculty (n=31); voluntary	Half-day workshop RE, PDT, BOT, FOR (FOR for more than half the workshop) (n=16)	Delayed and no training (n=15); training offered after the second rating	16 scripted videos; four weeks after training	IRR – ICC w mixed linear model (ICC = 0.40 pre & 0.43 post for RT; = 0.43 pre & 0.53 post control Log regression - no significant interaction b/w group and testing period (pre/post) p=0.88	Randomized, the high number declined randomization one-month delay between training and rating; no effect
Weitz et al. 2014 [24] Germany Single centre	Does RT improve the accuracy of assessment of physical examination skills? (five-point German grading code)	Randomized controlled to RT or none	Medical faculty (n=21)	90-minute workshop with in-person, video instruction and discussion (n=11)	No training (n=10)	242 students undergoing 10-minute physical exam skills assessment with a standardized patient	Reference rating using video-based reassessment of all 242 assessments using GRS and dimension-evaluation. Concordance between reference rating and faculty assessments. No effect of training on rating accuracy detected.	Randomized; small sample size; no effect on accuracy
George et al. 2013 [14] US Single centre	Determine type of FOR training for reliable and accurate use of assessment of surgeons using the Zwisch scale (four-point GRS)	Quasi-experimental Immersive vs accelerated training, non-randomized (depended on the availability of attending workshops)	Surgical faculty; voluntary (n=44)	Immersive – FOR with videos and practice testing, discussion Group workshop (n=34)	Accelerated one-hour Initial FOR definitions only; individual (n=10)	10 videos of real operations by staff and resident, rated immediately post RT	Proportion correct response for accuracy (80.2% immersive vs 88% accelerated); Spearman coefficient for correlation accuracy (0.90 immersive vs 0.93 accelerated); Cronbach’s for rater bias (0.045 immersive vs 0.049 accelerated).	No differences between the two types of training; cannot rule out underlying differences between the two groups; different scoring rubrics for two groups
Noel et al. 1992 [11] US Multi (12) centres	Determine the accuracy of faculty evaluations of residents clinical skills, how structured form and RT improve evaluations (structured form included four-point GRS)	Quasi-experimental; open form vs structured form vs. structured with training (allocation depending on when could attend, times for each random at each site)	Internists who serve as clinical evaluators; voluntary (n=203 total; 146 in groups 2 and 3)	15- minutes video on BOT, RET, PDT (n=69)	Structured form, no training (n=77)	Two scripted videos of resident history and physical on standardized patient; rated immediately	Accuracy scores (% correct) – no difference between RT and no training with structured form (64 vs 66% RT for case 1; and 63% vs 64% RT for case 2).	Looked at only structured form vs structured form with training for this review; structured form improved accuracy, training had small, non-significant improvements in some areas
Holmboe et al. 2004 [10] US Multi centre (16 programs)	Evaluate the efficacy of direct observation of competence training to change rating behavior (nine-point mini-CEX GRS)	Cluster designed randomized control trial; stratified, sealed envelopes; rated pre- and post-training	Internist faculty, nominated by program director, then voluntary (n=40) randomized (three lost)	Four-day course; “direct observation of competence” on day 2, included FOR, PDT and BOT (n=16)	No training (received same info packet as RT group) (n=21)	Nine scripted tapes (three cases at three levels of performance), rated eight months after training	Confidence intervals and range to estimate IRR (more stringent ratings in training group with smaller range). Regression showed significantly lower ratings.	High quality; positive effect; faculty had to be active in teaching for nomination; no differences at baseline ratings
Newble et al. 1980 [21] Australia Single centre	Value of RT on the reliability of scores & examiner selection for a clinical exam (standardized checklist with scores)	Randomized control trial, rated pre- and post-training	Surgical and internist faculty (n=18; nine of each, unsure how selected)	Limited 30-minutes training, individual, PDT (n=6); extensive two-hour group training, PDT & FOR with practice (n=6)	No training (rated two months later) (n=6)	Five videos of real students with standardized patient (students not aware standardized); rated a few days after RT	IRR Kendall’s co-efficient (pre-post scores: 0.48-0.44 intensive, 0.57-0.63 limited, 0.71-0.70 control); Spearman correlation between groups (0.8-0.9).	No effect of more intensive training on improving reliability; high training group most inconsistent at baseline (no difference between internists and surgeons); only thing that improved IRR was removing unreliable raters
Van der Vleuten et al. 1989 [17] US Single centre	Does training increase the accuracy of assessments of clinical skills (history & physical standardized checklists)	Randomized trial (three groups – doctors, med. students and lay people)	Physicians (surgeons and family doctor), prior examiners (physicians n=22)	FOR training with practice 1.5 hours (n=11)	No training (n=11)	Four tapes (two tapes of two cases) of real students with standardized patient, rated immediately	Accuracy % agreement with consensus scores (overall score 82% for RT and 81% control).	Only looked at attending cohort; all previous examiners; some mild improvements on individual cases, no significances or statistical comparisons given
Ludbrook et al. 1971 [20] Australia Single centre	Does examiner training decrease inter-examiner variability for the clinical skills exam	Randomized trial – not stated how or if for sure random; marked in pair (both trained or untrained)	Surgical faculty (n=16)	2.5 hour FOR training with videos, practice (n=16)	No training (n=16)	Medical school class, 100 students (each student in two cases with one pair examiners), marked one-week post- RT	Correlation coefficients between marking pairs (r=0.55 RT and 0.49 control within pairs p<0.01 for both; between pairs r=0.11 RT and 0.14 control p>0.05 for both).	No effect on correlation between rater groups; all had previous examiner experience; different scoring rubrics for two groups
Dudek et al. 2013 [19] Canada Multi-centre (four schools)	Does training improve the quality of ITERS for medicine residents (CCERR form used to assess ITER quality)	Randomized trial of five types of training – varying stages of feedback guide	Physicians who supervise medical trainees (n=98; only 37 returned at all required time points)	One CCERR score given (n=7), three scores given (n=6), one score + feedback given (n=9), three scores + feedback given (n=5)	No feedback (n=10)	Whatever ITERS they completed sent in; scores +/- feedback returned every six months x three	Mean CCERR scores - was improvement in scores for feedback groups, but was not significant.	Outcome of quality; only gave feedback and scores no true “rater training” workshop; low complete collection (37/98) Significantly underpowered (power calculation n=240)
Dudek et al. 2012 [18] Canada Multicentre (three sites)	Effectiveness of workshop at improving ITER	Uncontrolled pre/post-training design	Physicians who supervise trainees and complete ITERS; voluntary;(n=22)	Three-hours workshop (explain good FITER, recognize challenges)	None (pre/post-workshop)	ITERS of real clinical encounters pre and post-training	Mean CCERR scores (18.9 pre-training, 21.7 post p=0.02); ANOVA (no time interaction with CCERR items so changes were consistent pre/post for all items).	Outcome of quality

**Table 2:** Quality assessment of included studies in the scoping review

Reference	Abstract or summary	Randomization described	Blinding or concealment	Sample size calculation	N lost to follow up	Eligibility criteria given	Intervention described	Ethics approval	Baseline characteristics listed	Limits discussed	Quality and generalizability
Robertson et al. (2018) [15]	Yes	Yes	No	No	No	Yes	Well described	Yes	Yes	Yes	Moderate
Robertson et al. (2020) [16]	Yes	Yes	No	No	No	Yes	Well described	Yes	Yes	Yes	Moderate
Rogers et al. [13]	Yes	No	No	No	No	No	Briefly described	Not known	No	Yes	Poor; not generalizable
Spanager et al. [23]	Yes	N/A	N/A	No	No	Yes	Well described	Yes	Yes	Yes	Moderate
Cook et al. [22]	Yes	Yes	No	Yes	Yes	Yes	Briefly described	No	Yes	Yes, brief	Moderate; not generalizable
Weitz et al. [24]	Yes	Yes	No	No	No	Yes	Well described	Yes	Yes	Yes	Moderate
George et al. [14]	Yes	N/A	N/A	No	No	Yes	Well described	Not known	Yes, brief	No	Moderate
Noel at al. [11]	Yes	N/A	Yes	No	Yes	Yes	Briefly described	No	Yes, brief	No	Moderate
Holmboe et al. [10]	Yes	Yes (minimal)	Yes	No	Yes	Yes	Well described	Yes	Yes	Yes	High; generalizable
Newble et al. [21]	Yes	No	No	No	No	No	Moderately described	Not known	Yes, brief	No	Poor; not generalizable
Van der Vleuten et al. [17]	Yes	No	No	No	No	No	Moderately described	Not known	No	Yes	Poor; not generalizable
Ludbrook et al. [20]	Yes	No	No	No	No	Yes	Moderately described	Not known	Yes	Yes, brief	Poor; not generalizable
Dudek et al. 2013 [19]	Yes	No	Yes	Yes (not met)	Yes	Yes	Moderately described	Yes	No	Yes	Moderate generalizable
Dudek et al. 2012 [18]	Yes	No	Yes	No	No	Yes	Well described	Not known	Yes	Yes	Moderate generalizable
